# Inferior Vena Cava Filter Erosion Causing Symptomatic Obstructive Hydronephrosis

**DOI:** 10.1089/cren.2016.0070

**Published:** 2016-07-01

**Authors:** Nathan Locke, David Duchene, Priya Padmanabhan

**Affiliations:** Department of Urology, University of Kansas, Kansas City, Kansas.

## Abstract

***Background:*** Transcaval inferior vena cava (IVC) filter penetration involving the urinary tract is rare, but has been previously reported. We herein present unique management of symptomatic hydronephrosis secondary to erosion of an IVC filter limb into the lumen of the proximal right ureter.

***Case Presentation:*** A 59-year-old woman presented with abdominal and right flank pain in October 2015 and was found to have right hydronephrosis, apparently secondary to obstruction from erosion of an IVC filter limb into the proximal right ureter. This was effectively managed with percutaneous, endovascular, and endourologic procedures, without the need for a major invasive surgical procedure.

***Conclusion:*** Endovascular removal of the IVC filter was performed safely in this case and can be considered when the urinary tract is involved in filter erosion.

## Introduction and Background

Transcaval inferior vena cava (IVC) filter penetration is a well-recognized but often inconsequential complication of an IVC filter placement procedure. Involvement of the urinary tract requiring intervention, although rare, has been previously reported. We present a case in which symptomatic hydronephrosis secondary to erosion of a filter limb into the lumen of the proximal right ureter was effectively managed with minimally invasive techniques, without need for invasive surgical intervention.

## Presentation of Case

### Clinical history

A 53-year-old woman presented with a 1-month history of fluctuating abdominal and right flank pain. Medical history was significant for multiple prior abdominal operations, recurrent small bowel obstructions, and chronic abdominal pain. In addition, she had recurrent deep vein thrombosis (DVT) and noncompliance with anticoagulation, leading to the placement of a Cook Celect^®^ retrievable IVC filter (Bloomington, IN) in 2011. On initial presentation, she was afebrile and had normal vitals. Physical examination revealed mild diffuse abdominal tenderness and mild right costovertebral angle tenderness. Laboratory evaluation was largely unremarkable without leukocytosis or elevated serum creatinine. Microscopic urinalysis did not show pyuria or hematuria.

### Imaging

**FIG. 1.**
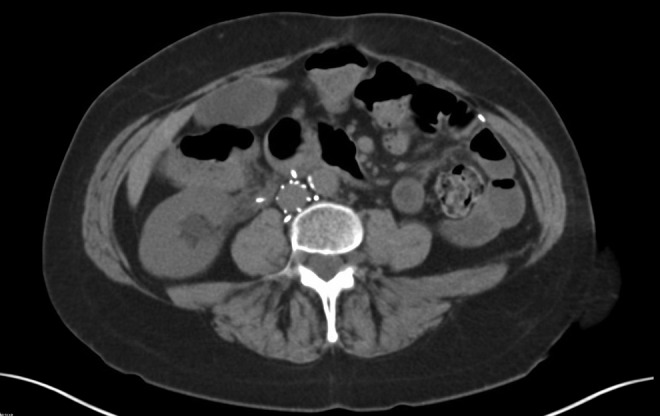
Axial cut CT showing erosion of multiple IVC filter limbs, including limb projecting into lumen of right ureter with proximal hydroureteronephrosis.

**FIG. 2.**
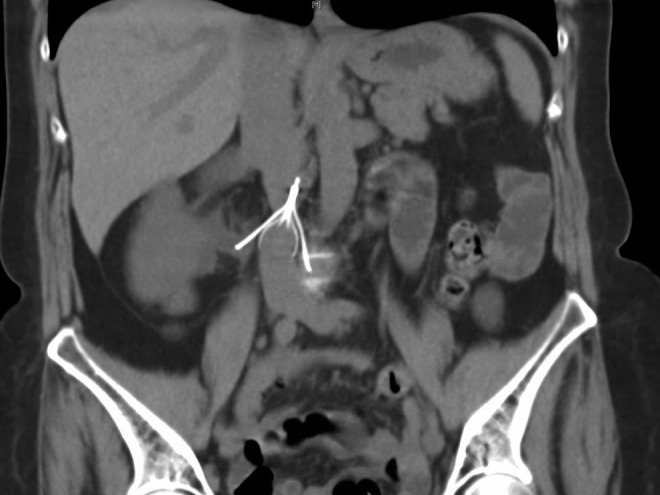
Coronal cut CT with additional demonstration of filter limb penetrating right ureter.

A CT of the abdomen and pelvis was obtained in the emergency room, which revealed moderate right hydronephrosis secondary to an eroded IVC filter limb projecting in the right proximal ureteral lumen (Figs. 1 and 2). Her complicated history had led to multiple previous CT scans, allowing for radiographic tracking of gradual migration of the filter limb into the ureter on sequential scans.

### Intervention

**FIG. 3.**
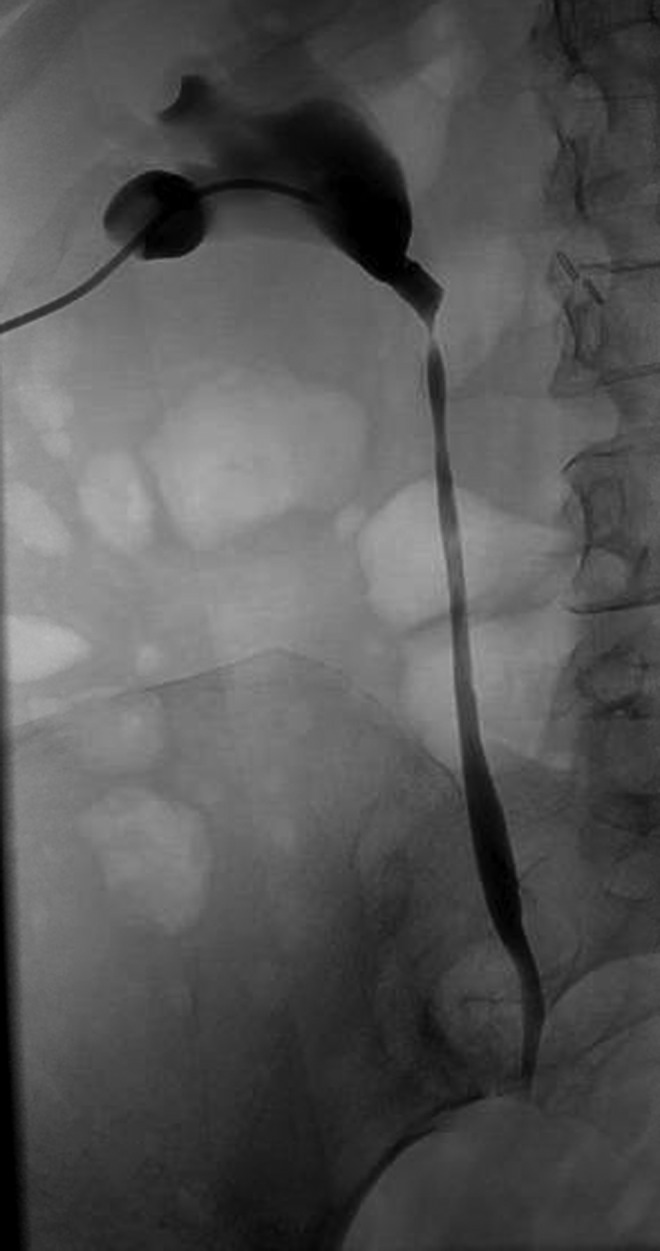
Antegrade nephrostogram immediately after filter retrieval showing luminal narrowing at site of limb penetration, without evidence of extravasation.

The patient was evaluated by the Urology, Vascular Surgery, and Interventional Radiology services. As there was no evidence of renal compromise, infection, hematuria, or hemodynamic compromise, no emergent intervention was performed. Review of her medical history revealed that her prior DVTs had occurred during previous periods of prolonged immobility. She was currently much more physically active. A hypercoagulability work-up was negative. Due to these factors and the symptomatic erosion of her filter, decision was made for retrieval of the filter. This was effectively performed through an endovascular approach by the Interventional Radiology service, using the right internal jugular vein for access. The filter retrieval hook was not embedded in the caval wall, which allowed it to be snared easily with a filter retrieval device. After this, the entire filter was withdrawn back into a sheath and removed. Concomitantly, percutaneous access for antegrade nephrostogram was performed (Fig. 3), which revealed some narrowing at the site of penetration but no contrast extravasation. A nephroureteral stent was effectively placed. Postprocedure course was uneventful and the patient was discharged home.

Two months later, a cystoscopy, retrograde pyelogram, and diagnostic ureteroscopy were performed to evaluate the ureter at the site of IVC filter tine erosion. Retrograde pyelogram and ureteroscopy showed improved but still mild narrowing at the site of injury. No urinary extravasation was noted. The percutaneous nephroureteral stent was exchanged for an indwelling ureteral stent. The ureteral stent was left in place for 1 month and then removed. Two months after ureteral stent removal, diuretic renography showed no evidence of obstruction.

## Discussion and Literature Review

Filter penetration is a well-recognized potential occurrence after IVC filter placement. Cook Celect^®^ filters are a relatively new model of retrievable IVC filter. These filters, made of a cobalt chromium alloy with platinum tungsten alloy radiopaque markers, are rated MRI conditional by the manufacturer, allowing for usage of MRI up to 3.0 Tesla. Interestingly, these filters have been noted to have an especially high rate of penetration, which increases with length of dwell time.^1^ Filter penetration can often be asymptomatic and alone is not considered an indication for filter retrieval, but complications can arise when surrounding organs are affected. Most commonly impacted is the duodenum, and involvement of the urinary tract is rare.^1^ Different management strategies have been used, and in some instances open surgical intervention has been indicated. In this case, the filter was effectively removed through an endovascular approach. No contrast extravasation was seen on postremoval inferior venocavogram. In one series, at this time published in abstract form only, extravasation occurred after 3% of complex filter retrievals. This was managed effectively with balloon tamponade and observation.^2^ Cook Celect filters have been retrieved with high rates of success in the past, as high as 96% in one series.^3^ Interestingly, this case highlights effective retrieval of a Cook Celect filter 1703 days after placement, which we believe is the longest reported dwell time before an effective retrieval of this filter model. A recently published case report described right hydronephrosis secondary to a misplaced IVC filter in the right gonadal vein. Open surgical intervention was required in this patient.^4^

## Conclusion

Ureteral injury from IVC filter penetration is a rarely noted complication. In select patients, IVC filter penetration involving the urinary tract can be managed with minimally invasive techniques without need for open surgical intervention.

## Abbreviations Used

CT: computed tomography
DVT: deep vein thrombosis
IVC: inferior vena cava
MRI: magnetic resonance imaging
